# Human Health Risk and Bioaccessibility of Arsenic in Wadis and Marine Sediments in a Coastal Lagoon (Mar Menor, Spain)

**DOI:** 10.3390/toxics13080647

**Published:** 2025-07-30

**Authors:** Salvadora Martínez López, Carmen Pérez Sirvent, María José Martínez Sánchez, María Ángeles Esteban Abad

**Affiliations:** 1 Department of Agricultural Chemistry, Geology and Pedology, Faculty of Chemistry, International Campus Mare Nostrum, University of Murcia, 30100 Murcia, Spain; melita@um.es (C.P.S.); mjose@um.es (M.J.M.S.); 2 Immunobiology for Aquaculture Group, Department of Cell Biology and Histology, Faculty of Biology, University of Murcia, 30100 Murcia, Spain; aesteban@um.es

**Keywords:** arsenic, mineralogy, bioaccessible fraction, risk, gastrointestinal phases

## Abstract

This study evaluates the potential health risks posed by geogenic arsenic in environments suitable for leisure activities, such as walking, bathing, and playing, for adults and children alike, as well as in neighbouring agricultural areas. The study includes an analysis of environmental characteristics and the main stream originating in the adjacent mining area, with water and sediment samples taken. The study area is representative of other areas in the vicinity of the Mar Menor Lagoon, which is one of the largest and most biodiverse coastal lagoons in the Mediterranean Sea. The general characteristics of the soil and water were determined for this study, as was the concentration of As in the soil and water samples. A granulometric separation was carried out into four different fractions (<2 mm, <250 µm, <100 µm, and <65 µm). The mineralogical composition, total As content, and bioaccessible As content are analysed in each of these fractions. This provides data with which to calculate the danger of arsenic (As) to human health by ingestion and to contribute to As bioaccessibility studies and the role played by the mineralogical composition and particle size of soil ingestion. The conclusions rule out residential use of this environment, although they allow for eventual tourist use and traditional agricultural use of the surrounding soils.

## 1. Introduction

Arsenic (As) has been a subject of controversy from ancient times to the present day [1]. It is considered a potentially hazardous element (PHE) with numerous effects on public health [2,3,4,5] and ecosystems [6,7]. The toxicity of arsenic (As) compounds depends on the chemical species with which they are associated and is ranked as follows: monomethylarsonous acid (MMA(III)) > dimethylarsonous acid (DMA(III)) > As triiodide (As III) > As pentoxide (As V) > monomethylarsonate (MMAV) > dimethylarsonous acid (DMAV) [8,9]. Ingestion can have various health effects, such as a decrease in red and white blood cells, skin changes, and organ irritation. Furthermore, it can lead to cancer, diabetes, cardiovascular disease, and reproductive problems [10,11]. Thus, this element is known to be carcinogenic, mutagenic, and teratogenic [12,13,14]. Additionally, compounds containing it have been classified as Group I carcinogens by the US Environmental Protection Agency [15] and the International Agency for Research on Cancer [16]. The toxicity of this metalloid is contingent upon a number of variables, including its overall concentration, its potential solubility, its leachability, its bioavailability, and its capacity to be released from different matrices [17,18,19].

Bioaccessibility is defined as the fraction of a contaminant that is soluble in the gastrointestinal tract and available for absorption. This can be calculated by in vitro simulation. This definition is based on a comprehensive literature review [15,20,21,22,23,24,25,26].

The term “bioavailable fraction” is used to refer to the fraction of the contaminant that is absorbed by the intestinal epithelium. To assess the potential risk posed by this contaminant, it is important to assume that the entire ingested dose is bioaccessible. It is crucial to avoid overestimating the risk at a given site, since there may be no actual risk present [15,24,27,28,29,30]. The observed differences in the bioavailability of metals in mine materials under simulated gastrointestinal tract conditions suggest that the solubility of each metal is contingent upon its chemical speciation and its capacity to bind to different soil and sediment materials [31,32]. In the context of risk studies of exposure to this trace element, it is critical to comprehend not only the bioaccessibility of the element in question but also its dynamics in the soil and its association with mineralogical phases. The mobilisation and bioaccessibility of As in soil are influenced by a number of factors, including chemical species, pH, redox potential, the presence of manganese and iron oxides, phosphate, soil texture, and clay minerals, as well as adsorption on carbonates and organic matter [33,34,35]. The presence of secondary iron minerals, such as goethite, jarosite, and schwertmannite, facilitates the sequestration and retention of the element, thereby promoting a natural attenuation process in soils [36]. Consequently, it is of paramount importance to assess the behaviour of this element in the context of diverse environmental conditions.

The employment of statistical procedures is a common resource that facilitates the interpretation of the results. Depending on the number of samples and the variables considered, different options can be used to better understand the problem. A plethora of strategies have been employed to establish a correlation between bioaccessibility and various variables. These include bivariate correlations, as well as the utilisation of machine learning algorithms. Other strategies include the application of Principal Component Analysis and regression [37,38].

The initial phase of this investigation should focus on the concentration, with other parameters being required for decision-making purposes [39]. This is particularly important in the case of mining soils, given their geogenic characteristics [40,41]. The importance of mineral associations, different alteration states, and sorption/desorption processes in the gastrointestinal bioavailability of metals has been established by numerous studies [42]. Higher values of bioaccessible As are found in materials that have undergone low alteration and are rich in primary minerals, compared to materials that have undergone significant supergene alteration and have been transported by runoff, wadis, etc. [43].

In addition, the particle size of the sediment must be taken into consideration. The size of soil fractions is an important aspect to consider. In vitro tests generally recommend the use of the 250 μm fraction, as it is considered to be the most adherent to children’s hands [44,45] and is the official standard size. The DIN protocol, conversely, utilises the particle size fraction of ≤2 mm, categorised as fine soil. The <150 μm fraction is advocated in Method 1340 [46,47] for the dermal route, and alternative studies have recommended even more diminutive fractions (e.g., <63 μm or <50 μm) [48,49,50].

The objective of this analysis was to evaluate the potential risks of human exposure by ingestion, along with the associated uncertainties (mineralogy and granulometry) in the soils of the Rambla del Beal Catchment and its outflow into the Lo Poyo Wetland (Mar Menor Lagoon, Southeastern Spain). Furthermore, the various modes of transport of As in the wadi, either in soluble or particulate form, and its impact on the beach of Los Nietos have been examined. This analysis comprises a preliminary study of arsenic concentration in beach sediments within the Coastal Guidelines 2021 [51] sediment management programme (GEOSEM-ThinkInAzul).

## 2. Materials and Methods

### 2.1. Study Area

The study area is the Beal wadi, situated southeast of Campo de Cartagena (SE Spain). It is one of the drainage routes of the Sierra Minera de Cartagena, which flows into the Mar Menor Lagoon. The area is close to a wetland (Saladar de Lo Poyo) that has the category of a Natural Park, Site of Community Importance (SCI), and Special Protection Areas for Birds (SPA). Additionally, it hosts a tailings pond at the mouths of the Beal and Ponce wadis. The levels of As in this area can exceed 300 mg·kg^−1^, and its bioaccessibility can be high [39]. The Beal wadi suffers hydrological extremes of persistent floods and droughts due to its semi-arid climate. It remains dry for months or years, but when torrential rains occur, it collects the drainage from the mining area, giving rise to flash floods that discharge into the Mar Menor Lagoon.

**Figure 1:**
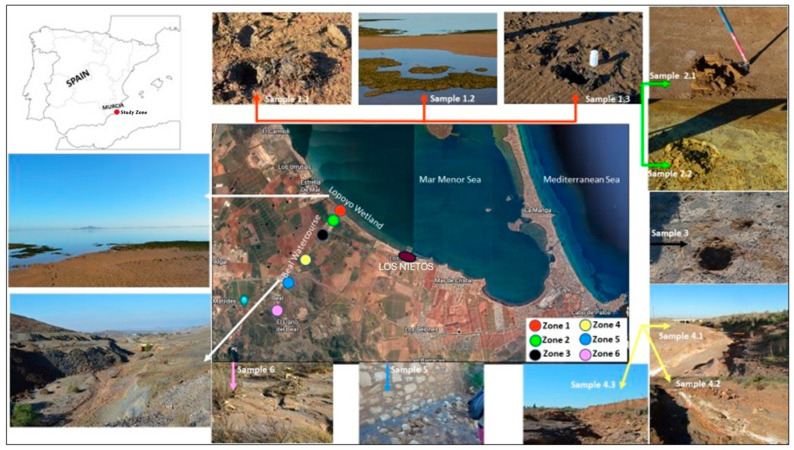
Localisation zone.

Los Nietos Beach is located near the mouth of the Beal wadi (Figure 1), and it is important to study the possible arsenic contamination, due to the possible mining influence of the marine sediments.

### 2.2. Materials

Six sampling areas were selected in the river course of the Beal wadi, in order to carry out the characterisation of the zone. A total of 11 soil samples were collected (Figure 1), The samples were taken from the top 25 cm (arable layer), thus collecting around 2–3 kg of sample at each sampling point. The collected samples present different characteristics and structures, as can be seen in the photographs in Figure 1, due to their provenance in a mining waste basin. The sampling areas were:

Zone 1: The wadi mouth (samples: 1.1, 1.2, and 1.3). They correspond to Arenosols, beach soils.

Zone 2: The cone of the mouth of the wadi leading to the sea (samples: 2.1 and 2.2). Area of direct discharge of mine sludge modified by the activity of the wadi over the years.

Zone 3: The last stretch of the wadi before reaching the Lo Poyo Wetland area (sample: 3). Part of the riverbed representative of the mixture of direct discharges with the eroded materials contributed by the wadi.

Zone 4: The middle zone of the wadi bed (samples: 4.1, 4.2, and 4.3). They are fluvisols that have formed at the expense of the neighbouring soils: luvic xerosols and xeric luvisols.

Zone 5: The middle area of the wadi bed, near the urban centre of El Beal (sample: 5). Fluvisols formed with eroded materials from Sierra Minera.

Zone 6: The area at the head of the wadi (sample: 6). Fluvisols formed with eroded materials from Sierra Minera.

Each soil sample was sieved at a different granulometric fraction to obtain four subsamples (<2 mm, <250 μm, <100 μm, and <63 μm) studied as if they were independent samples, so a total of 44 samples were used.

The fraction <2 mm is the part that is considered fine soil and is the standard for analytical characterisation of a soil, the fraction <250 μm is used to calculate bioaccessibility, and the fraction <100 μm represents a sandy soil formed by grain selection processes and is common in beach sand. Finally, the fraction <63 μm represents the materials that are suspended in seawater when the wadi discharges.

Surface water was sampled at three points after a rainfall event. Water samples W-1 (zone 1), W-2 (zone 6), and W-3 (near zone 4) correspond to water from the wadi at the mouth, water from the wadi at the headwaters, and water from puddles in the unaffected area, respectively.

Twenty sampling points were selected at Los Nietos Beach for As determination. Four fractions of each marine sediment (<2 mm, <250 μm, <100 μm, and <63 μm) were studied as if they were independent samples.

### 2.3. Methodology

#### 2.3.1. Characteristics of the Soil, Marine Sediments, and Waters

Soil samples were air-dried and sieved through a 2 mm sieve for general analytical determinations. The pH and electrical conductivity (EC) were determined in a 1:5 (weight/volume percent) suspension of soil in pure water.

The organic carbon content was determined by sulphochromic oxidation, according to the NF ISO 14235 standard [52].

The granulometric analysis of the soil samples was carried out after dispersion in a hexametaphosphate solution. The particle size distribution was evaluated by a static light scattering (LS) instrument equipped with a microliquid module (Coulter LS 13320, Brea, CA, USA). Soil texture is expressed according to the FAO soil description guide [53] as clay <2 μm, silt 2–63 μm, and sand >63 μm.

The various particle size fractions examined in this study were obtained through mechanical sieving using approved sieves.

Surface waters were filtered through 0.45 μm pore size filters and properly stored, including refrigeration at 4 °C for analytical determinations. The pH, redox potential (Eh), and EC were determined by the usual procedure.

The results showed RSDs close to 5% and agreed with the certified values. All reagents were of analytical grade or Suprapur quality. Stock standard solutions were Merck Certificate AA standards. Pure water was used in all the experiments. Plastic and glassware were cleaned by soaking in a 14% (*v*/*v*) HNO_3_ solution for 24 h and then rinsing with Milli-Q water. Spikes, duplicates, and reagent blanks were also used as a part of our quality assurance/quality control.

#### 2.3.2. Determination of As

- Determination of the total As content

The total As content in the grain size fractions (<2 mm, <250 μm, <100 μm, and <63 μm) was determined for each of the samples collected.

To determine the total trace element content, the samples were first ground to a fine powder using a zirconium ball mill. Aliquots (0.1 g) were placed in sealed PTFE containers, and a mixture of 5 mL concentrated hydrofluoric acid, 200 mL concentrated nitic acid, and 5 mL pure water was added. After digestion in an ETHOS laboratory microwave system (Milestone, Sorisole, Italy) equipped with temperature and pressure feedback controls, operating at a maximum power output of 1500 W, the obtained solutions were transferred to a volumetric flask and brought to 50 mL. Arsenic concentrations were determined by electrothermal atomisation atomic absorption spectrometry (ETAAS) using a CONTRA AA700 high-resolution continuous source atomic absorption spectrometer (HRCS-AAS) from Analytical Jena AG, Thuringia, Germany. The reliability of the results was tested by analysing certified reference materials (NIST SRM 2711 Montana Soil and NIST SRM 2709 San Joaquin Soil).

On the other hand, after three rainfall episodes occurred in a year, a total of nine runoff water samples were collected in the wadi and depressed areas with low drainage. Surface waters were filtered through 0.45 μm pore filters and properly stored and refrigerated at 4 °C until the determinations that were carried out using the same analytical technique.

In addition, a non-chromatographic speciation analysis was also carried out on the water samples for As (III) and As (V) through a procedure adapted from El-Hadri et al. [54] and Cava-Montesinos et al. [55]. The procedure consisted first of directly measuring the signal obtained by hydride generation atomic fluorescence spectroscopy of the unreduced sample (only As (III) originates hydride) and then measuring the total As after reduction of the As (V) present in the sample with KI. The As (V) concentration was obtained from the difference between the two readings. In this environment, inorganic forms As (III) and As (V) are the main water-soluble species [56]. The arsenic concentration was determined using atomic fluorescence spectrometry with an automated continuous flow hydride generation (HG-AFS) spectrometer (PSA Millennium Excalibur 10055, P S Analytical, Kent, UK). The experimental conditions and instrumental parameters used were as follows: wavelength (nm) = 197.3, primary current (mA) = 27.5, boost current (mA) = 35, delay time (s) = 15, analysis time (s) = 30, memory time (s) = 30, and air flow rate (mL/min) = 300.

The detection limit (DL) for the determination of total As in soil, water, and chemical extraction was 0.5 μg L^−1^, and the quantification limit for As in soil was 0.05 mg kg^−1^.

- Determination of As bioaccessibility and Extraction in the gastrointestinal tract.

In vitro extraction methods were used for this determination. There are several in vitro methods to assess the relative bioavailability of metals, but in this study, the method used was that of the Solubility Bioaccessibility Research Consortium (SBRC), as it provides an adequate relationship between the bioavailability and bioaccessibility of As and a high correlation with the results obtained in the in vivo experiments [57].

The determination was carried out according to the standard operating procedure (SOP), the two phases distinguished representing the stomach and intestinal.

**Stomach Phase (S.P.).**

The fraction normally used to assess bioaccessibility is <250 μm, due to the higher probability of direct contact with humans [58,59]. In this study, three other particle size fractions (<2 mm, 100 μm, and <63 μm) were also studied.

For the extraction, 1 g of the soil samples was weighed into 250 mL plastic bottles, and 100 mL of 0.4 M glycine was added. The pH of the suspensions (1.5 ± 0.5) was adjusted with concentrated HCl before they were placed in a bath shaker (27L OVAN bath, OVAN, Barcelona, Spain) at 37 °C shaking to simulate the digestive process taking place in the stomach. After one hour, 20 mL of the suspension was withdrawn with a syringe and filtered through a 0.45 μm nylon microfilter. Teflon or other suitable plasticware was used for handling these liquids.

**Intestinal Phase (I.P.).**

For the intestinal phase, the remaining stomach phase suspensions were used. The pH was raised to pH 7 ± 0.5 with 50% (*v*/*v*) NaOH before adding 175 mg of pork bile salts and 50 mg of pancreatin. The samples were returned to the bath at 37 °C and shaken for a further four hours, simulating passage through the small intestine. The resulting suspension was extracted through a 0.45 μm nylon microfilter and acidified with HNO_3_. Teflon or other suitable plasticware was used for handling these liquids.

#### 2.3.3. Mineralogical Composition

A semiquantitative estimation of the mineralogical composition of the samples was made by powder X-ray diffraction (XRD) analysis using Cu-Ka radiation with a PW3040 Philips Diffractometer (Amsterdam, The Netherlands). X-powder 12 software was used to analyse the X-ray diffractograms obtained [60]. The powder diffraction file (PDF2) database was used for peak identification.

#### 2.3.4. Analysis for the Development of Cartography: Laminar Erosion and As Concentration

For the cartography of the As contents of the study area, information from different sources [39,61,62] was used to obtain the resulting raster that best fits reality.

The platform chosen for most of the procedures and tests described below was ArcGIS 10, which is an effective geographic information system (GIS) suited to spatial analysis tasks that has a module of tools that allow capabilities to be added, performing tasks like geo raster processing, modelling, and spatial analysis.

Laminar soil erosion mapping was carried out on the basis of data published in the National Inventory of Soil Erosion (2002–2022) [63]. An arithmetic overlay raster analysis was then performed based on the combination of 2 layers:

Laminar and runoff soil erosion rate (t/ha/year) in this basin.

As content (mg kg^−1^) for the studied hydrological basin.

#### 2.3.5. Risk Analysis

The initial step in conducting a human health risk analysis study was to determine the daily doses of exposure to the pollutant, which represented the magnitude of exposure and were expressed in units of mass of contaminant exposed per unit of body mass per day. To calculate this, it was necessary to determine the concentration of As in the soil and its bioaccessible fraction. This was conducted for the oral exposure route, in accordance with the methodology of the United States Environmental Protection Agency [15]. The results were obtained from bibliographic information and various databases,

Highly conservative input parameter values, representative of the worst-case scenario, were used for the exposure assessment, so the results are considered to reflect safe levels of health protection for the receptors considered. The receptors considered for the different potential land uses were adults (agricultural workers, residents, and tourists) and children (both residents and tourists). Children are not included for the agricultural use scenario.

- **Calculation of the Oral Chemical Daily Intake (CDI)**

Dust/soil ingestion is a very important route of exposure to environmental contaminants. This is considered to have a higher probability of occurrence, since it is linked to the accidental ingestion of soil and is therefore considered to be the main exposure risk route in risk analysis studies of potentially contaminated soils. Chemical Daily Intake (CDI) was calculated with the equation provided by the EPA [64], and the parameter values are summarised in Table 1:

$$CDI\;\left(Ing\right)=\;\frac{mg}{kg.day\;}\;\frac{Cs\;\times \;IR\;\times \;EF\;\times \;ED\;\times \;CF\;\times \;FI}{BW\;\times \;AT}$$

where Cs is the concentration at the point of exposure (As mg kg^−1^); IR is the soil ingestion rate (mg/day), EF the exposure frequency (day), ED the exposure duration (year), CF the unit conversion factor of 10^−6^ (dimensionless), FI the ingest factor (dimensionless), BW the body weight (kg), and AT the average time (days). Risk/Hazard characterisation. At this stage, the risk or hazard was characterised. This quantification will be the basis for decision-making in future reclamation projects in this area affected by past mining activities.

- **Risk characterisation for carcinogenic pollutants.**

Risk, defined as the incremental probability of developing cancer over a lifetime, was characterised using the risk equation shown below to calculate the risks for carcinogenic pollutants such as As [65]:

Risk=CDI × SF

CDI: Chemical Daily Intake (mg/kg·day) and SF: carcinogenic potency factor or slope factor (mg/kg·day) (Table 2).

Risk > 10^−5^: an unacceptable health risk is considered to exist, and mitigating measures should be taken.

Risk < 10^−5^: the risk is acceptable.

- **Hazard characterisation for non-carcinogenic contaminants.**

In this step, the hazard is characterised for non-carcinogenic contaminants. The hazard index (HI) relates the exposure dose to the reference dose for the exposure route and the corresponding exposure period. The hazard characterisation was performed using the following equation:

$$HI=\frac{CDI}{RfD}$$

RfD: Reference dose (mg/kg·day) (Table 2 summary of toxicological information on As). Hazard-based:

HI > 1: an unacceptable health risk is considered to exist, and mitigating measures will have to be taken.

HI < 1L the risk is acceptable.

#### 2.3.6. Statistics

The data were treated using IBM SPSS statistics v. 20 and Minitab 17. Correlations between the different variables were carried out in order to study the dependence relationships that exist among the variables determined. In addition, a Principal Component Analysis (PCA biplot) was performed to study the contribution of each factor in the samples evaluated.

## 3. Results and Discussion

### 3.1. General Analytical Features

Table 3 shows the analytical characteristics of the soils. The pH range along the channel is highly variable, ranging from very acidic (2.8) to basic (7.7). Zone 1, which corresponds to the mouth of the wadi, has soils with a moderately basic pH that is associated with the presence of carbonates.

The pH level of the soil may contribute to the natural attenuation of contaminant dispersion. When the pH is basic, trace elements tend to fix in soils, resulting in a low degree of leaching. This parameter is of great importance, as it directly affects the mobility of arsenic (As). This metalloid is soluble at acidic (pH < 4) and basic (pH > 8) pH levels, enabling soils at higher risk to be identified in advance (ref). However, the remaining soil samples exhibit acidic or slightly acidic pH values, which may be linked to the spontaneous oxidation of sulphide minerals releasing more As into the environment. The soil in the area exhibits a high degree of salinity, which is a result of past mining activities and the presence of soluble salts in varying concentrations.

The lack of vegetation in the area can be attributed to the extreme pH and salinity conditions, which have led to a low organic matter content in most of the soils studied. The influence of organic matter content on As mobilisation can be conceptualised through two distinct mechanisms. Firstly, it has been demonstrated that organic matter can facilitate the formation of organic complexes with highly toxic As. Secondly, the presence of organic matter has been shown to enhance reducing conditions by increasing the concentration of As(III), which is recognised as the more toxic form in comparison to its more oxidised forms. The values of the samples from sampling zone 1 may be associated with organic residues from the sea, as well as algae and marine debris.

The predominant texture of the samples is sandy or sandy loam, with the exception of the samples from zones 4 and 2, which contain a higher percentage of clay.

### 3.2. Mineralogical Composition

The mineralogy of the soils studied indicates the diverse origins and processes of the materials transported by the wadi. Three distinct groups can be identified: the first comprises materials derived from mining activities, including clinochlore; greenalite; goethite; and secondary alteration products (jarosite, akaganeite, etc.). The second group is composed of materials from the surrounding soils, which include illite, quartz, calcite, and gypsum. The third group, located at the mouth of the wadi (zone 1), exhibits characteristics of both the aforementioned groups, with aragonite present, suggesting a marine biotic influence. However, the absence of arsenopyrite and other arsenates indicates that the source of arsenic is not attributable to these minerals. Instead, the presence of pyrite, secondary iron sulphide alteration minerals, and phyllosilicates suggests that the arsenic originates from these sources [62,66].

In sampling areas 2 and 3, the predominant mineral is natrojarosite, which is of supergene origin and results from the oxidation of pyrite. It occurs in association with various polyhydrated sulphates, forming efflorescences and identifying copiapite, bianchite, and butlerite, among other materials. These are products of the concentration process of the mined metal ores that have been discharged into the wadi.

Sampling area 4 can be considered as the reference material from the primitive wadi bed, illustrating the mineralogy of the red soils that cross the wadi. These soils have a high content of clay minerals such as illite and clinochlore, calcite, and gypsum. These soils exhibit a fluventic character, indicative of materials eroded from higher levels that have previously undergone supergene alteration processes within a primitive gossan formed in the mining area. Consequently, the As content of these soils is lower than in the other soils studied but higher than the background value given for the area [67]. The influence of nearby volcanic outcrops is evidenced by the presence of amphiboles.

Sampling areas 5 and 6 demonstrate the outcome of the combination of eroded materials from the mining saw with the ore washing sludge deposited in the wadi.

**Figure 2:**
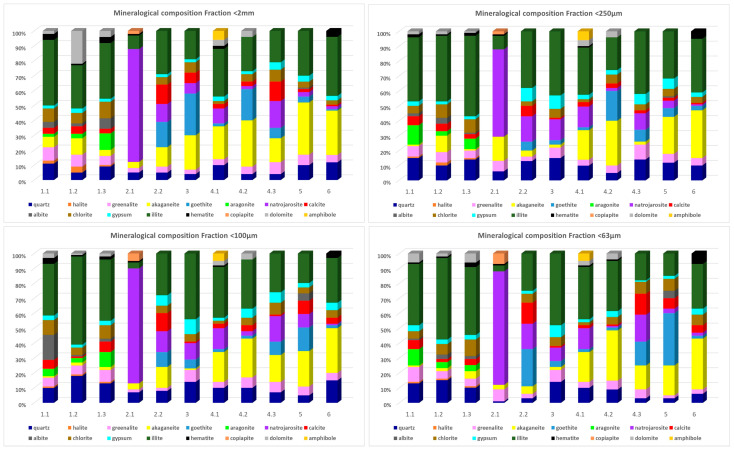
Mineralogical composition of Rambla del Beal.

The minerals that contribute heavy metals to the bioaccessible fraction include carbonates, iron oxyhydroxides, and soluble sulphates. Figure 2 displays the mineralogical composition based on the particle size fraction. The same minerals are present in all four fractions that were studied. However, it is important to note that the proportion of certain minerals varies depending on the particle size. The most significant differences are observed between fractions <250 µm and <63 µm. The coarser fraction exhibits higher concentrations of Fe hydroxides and a lower proportion of phyllosilicate minerals.

**Figure 3:**
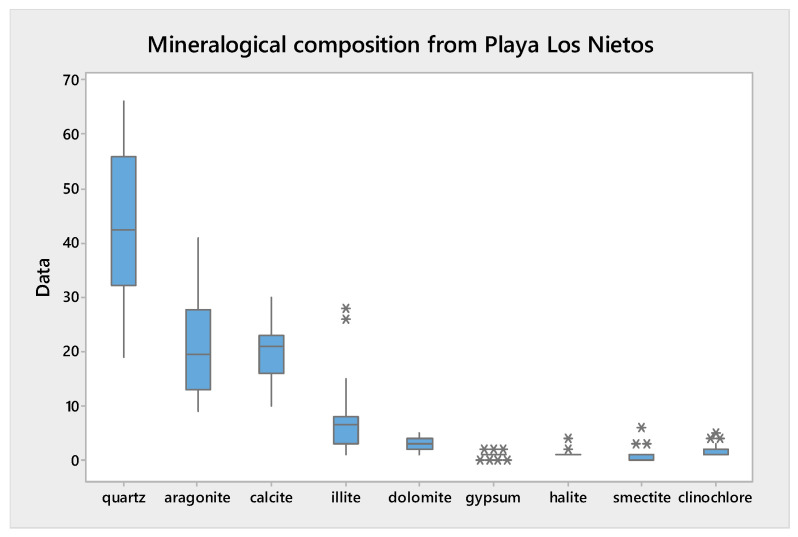
Summary of the mineralogical composition from Los Nietos.

The marine sediments near the mouth of the wadi exhibit a mineralogy similar to group two, influenced by the sea. The most abundant minerals are quartz, aragonite, illite, and calcite. There are no traces of minerals related to the mines, such as jarosite and greenalite. Figure 3 summarises the mineralogical composition of the studied samples.

### 3.3. Arsenic Content

#### 3.3.1. Arsenic in Particulate Material

In order to carry out this research, a study was previously carried out to determine the mobility of arsenic in the hydrological basin formed by the Beal and Ponce wadis. The arsenic mobilisation map obtained is shown in Figure 2 [61]. The erosion that occurs in the study zone is high, and the amount of arsenic that is mobilised and that may be entering the Mar Menor Lagoon is significant. This is the reason why it was decided to carry out this exhaustive analysis of the Beal wadi and determine the real risk to people’s health.

**Figure 4:**
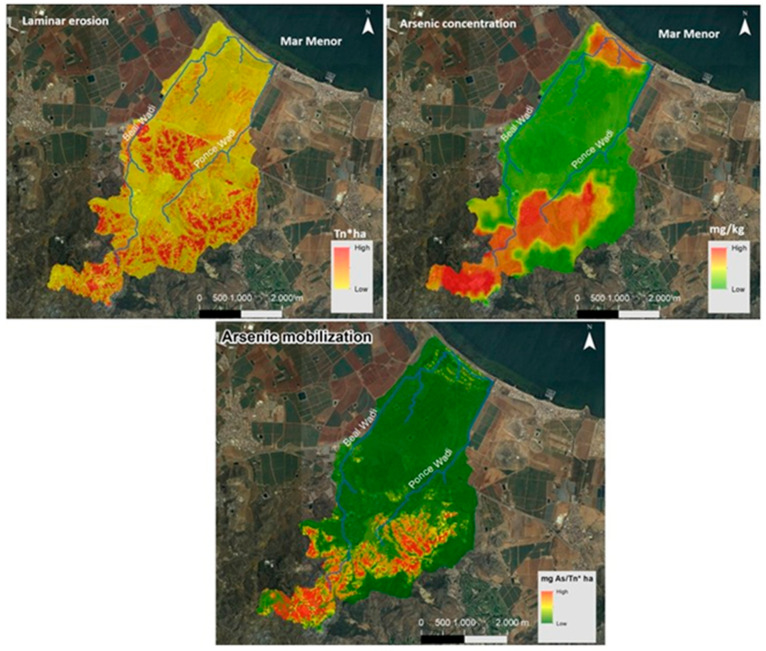
Arsenic mobilisation map.

Laminar erosion in the headwaters of the wadi is significant, with values of approximately 200 t·ha^−1^·yr^−1^, indicating a high mobilisation of materials. These sediments are deposited in the bed of the wadi as the slope decreases, but the rates of laminar erosion remain high, exceeding 100 t·ha^−1^·yr^−1^, as shown in Figure 4.

Based on the rate of laminar erosion and the concentration of As at the mouth of the Beal wadi, it is estimated that 5 t·ha^−1^·yr^−1^ of materials contaminated with trace elements enter the Mar Menor Lagoon. Additionally, a map of arsenic mobilisation in the study area was obtained (see Figure 4).

A juxtaposition of the As concentration and mobilisation maps unequivocally demonstrates the absence of mobilisation in the coastal zone, despite the fact that the As concentration is comparable to that observed in the mining zone. The phenomenon under discussion can be attributed to the fact that the material that is observed in the vicinity of the sea is a material that has been transported by anthropogenic activity. It has not been eroded by natural processes and exhibits a low natural solubility.

However, the material corresponding to zones 2 and 3 is susceptible to yield. This is due to the presence of acidic waters, reducing conditions, and supergene alterations in oxidising conditions, as is the case in other environmental conditions.

#### 3.3.2. Total Arsenic

The results of the analysis conducted on four soil fractions (i.e., <2 mm, <250 µm, 250–100 µm, and <63 µm) are presented in Table 4. In the study area (Beal wadi), the As concentration exhibited a considerable range, from a minimum of 75 mg/kg to a maximum of 1380 mg/kg. The highest concentration was observed in the upper part of the wadi bed (Zone 6).

**Figure 5:**
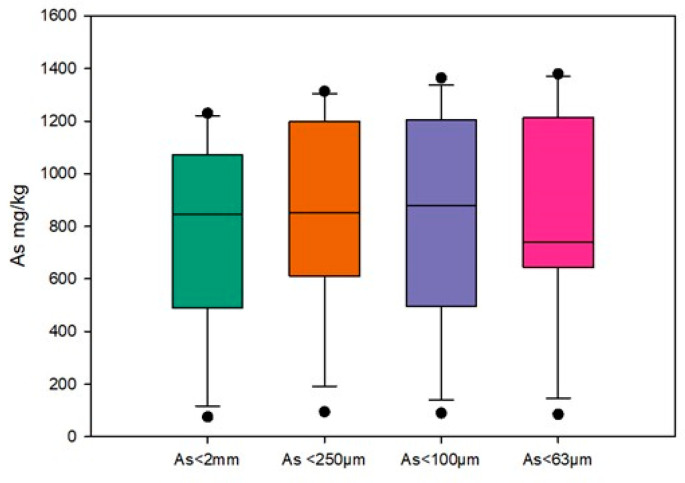
Total arsenic in the soils (mg/kg).

Figure 5 presents the mean values and ranges obtained using a box and whisker plot. The mean concentration in the <63 µm fraction was 861.3 mg/kg, representing the highest mean concentration of the four particle size fractions studied.

The lowest mean concentration was determined in the <2 mm fraction, where it was 74 mg/kg. The results demonstrate the influence of particle size on the total As content in the different fractions. The higher As accumulation observed in the finer particles may be associated with their larger surface area per unit mass.

In the study area (Los Nietos Beach), the As concentration exhibited a range from a minimum of 9 mg/kg to a maximum of 128 mg/kg in the <2 mm fraction. In the rest of the fractions studied, the As concentration determined was higher, ranging from a minimum of 11 mg/kg to a maximum of 44 mg/kg in the <250 µm fraction, 18–222 mg/kg in the <100 µm fraction, and 22–250 mg/kg in the <63 µm fraction. According to the As concentration determined in the <2 mm fraction, in this work, a classification of the studied material was made following the Dredged Material Characterization Guidelines (2021) [51], in cases where these materials were dredged, and looking for a productive use of the materials, the results show that 75% of the studied materials can be used as sand for beach regeneration. The rest of the materials studied are classified in categories B and C, so they require decontamination techniques to be used as sand for beach regeneration.

#### 3.3.3. Soluble Arsenic

The water samples correspond to surface waters remaining after rainfall. Table 5 shows that the As content of the water samples was generally low. The highest concentrations were found in those with the lowest pH (acid mine drainage), waters that accumulate in poorly drained places, where they temporarily accumulate after rainfall. They corresponded to the highest part of the wadi (point 6). Table 5 also shows the results obtained for arsenic speciation. The pentavalent form of As predominated over the trivalent form in surface water, which is more oxygenated than groundwater. The concentration of As (III) was very low in these waters and was higher in those with an acid pH, the As (V) concentration representing more than 95% of the total As content in the water.

#### 3.3.4. Bioaccessible Arsenic

To evaluate the relative bioaccessibility factors for each of the four fractions studied, data showing the highest value in the simulation phases of the gastrointestinal tract were employed. The concentration in the stomach phase (As A) was found to be significantly higher than the concentration determined in the intestinal phase (As N) for all four soil fractions, as shown in Table 6. Arsenic has a higher solubility at an acidic pH, which is the case in the stomach phase. The mean concentration in the stomach phase was determined in the following order: [fraction < 250 µm] > [fraction < 2 mm] > [fraction < 63 µm] > [fraction < 100 µm].

The highest concentration of As bioaccessible in the fraction with a diameter of less than 250 micrometres (µm) was found in samples 1.2, 1.3, and 4.1, with concentrations of 19.5, 17.8, and 18.1 mg/kg, respectively. The fraction with a diameter of less than 2 mm showed the highest concentration of As in the same samples as the previous fraction. The concentrations in the <250 µm fraction were 18.3, 16.1, and 15.3 mg/kg, respectively, for samples 1.2, 1.3, and 4.3. Sample 1.2 exhibited a high content of carbonates (33%) and an average content of iron minerals (20%), while sample 4.3 exhibited an average high content of iron minerals (48%) and an average content of 18% of carbonate minerals.

The highest concentrations in the intestinal phase were found in samples 1.1 and 4.2 (3.5 mg/kg As) in the <63 µm fraction (Table 4). Sample 1.1 exhibited a high content of carbonates (approximately 27%), while sample 4.2 displayed a high content of Fe minerals (approximately 44%), which may contribute to the bioaccessible fraction.

**Figure 6:**
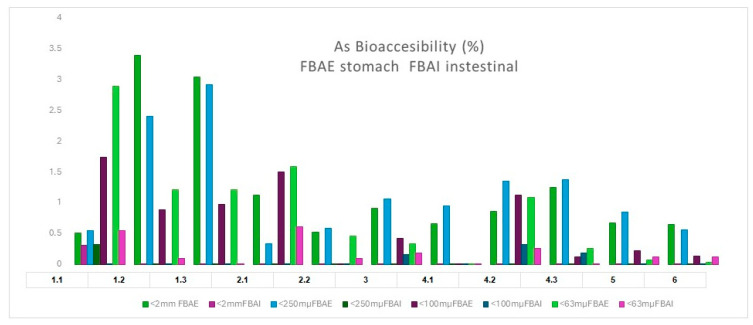
As bioaccessibility (%) soils.

Figure 6 illustrates that the percentage bioaccessibility of As was higher in the stomach phase than in the intestinal phase. Samples 1.2, 1.3, 4.2, and 4.3 exhibited the highest values. Despite exhibiting the highest total As content, zone 6 displayed the lowest percentage in both the stomach and the intestinal phases.

**Figure 7:**
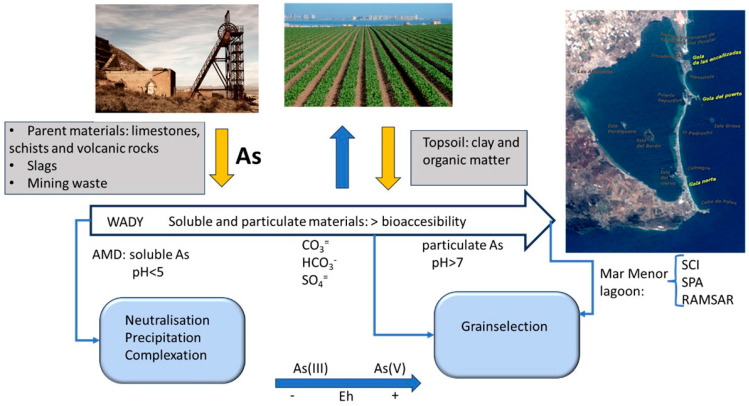
Main processes taking place in the Beal wadi.

The relationship between these soils and carbonate content is evident, as observed in previous studies [29,39]. The carbonates present in the wadi neutralise soluble As by immobilising it. However, in an acidic stomach environment, it is released, leading to an increase in bioavailability. Furthermore, organic matter increases the bioavailability of the element, as evidenced by the samples from zone 1 [39]. Figure 7 provides a synopsis of the various aspects discussed in this work, highlighting the principal scenarios to be considered in a comprehensive risk analysis process derived from the presence of the element.

When water is present, processes such as the transport of particulate and soluble material, dissolution, hydrolysis, redox reactions, complexation, carbonation, and precipitation can take place in the wadis that start in the elevated areas of a mining area. Once the rainy season ends and surface water disappears, the remaining sediments in the riverbed undergo secondary processes that result in the formation of unique mineral species, such as the efflorescence of hydrated sulphates, carbonates, and oxyhydroxides. This is due to the action of pore water, which can remain in the sediments of the wadi bed for a long period of time. The wadi water in the upper reaches has an acidic pH (see Table 5), which is neutralised in the lower reaches and at the wadi mouth. These neutralisation reactions occur in the middle part of the wadi and are caused by the presence of calcium carbonate in the soil. These reactions result in the precipitation of Fe(III) oxyhydroxides (which may include As in the crystal lattice) and calcium arsenate and the disappearance of any reduced As species if the medium is oxidising, as determined by the Eh values. In the event of torrential rain, neighbouring soils may flood and become enriched with the ETPs carried by the wadi water. It is a diagenetic, non-anthropogenic process. The presence of high concentrations of organic matter leads to the formation of organic compounds containing arsenic (As), which increases the environmental risk and favours reductive conditions.

### 3.4. Chemical Daily Intake and Risk Characterisation for Public Health

Firstly, the concentration of arsenic in the fractions <2 mm, <250 µm, <100 µm, and <63 µm was employed to calculate the CDI dose. Subsequently, to calculate the exposure dose, taking into account the bioaccessibility factor, the bioaccessibility content of arsenic in the same fractions was utilised. To characterise the risk, the carcinogenic risk slope (1.5) and the RfD (0.0003) for the hazard index were applied. Table 6 and Table 7 present the exposure assessment and risk characterisation for the carcinogenic risk and the hazard index for children and adults, respectively.

In scenario E1 (residential use), all studied samples presented an unacceptable level of risk for children (Table 6) and adults (Table 7) when the CDI and carcinogenic risk were calculated for the four particle size fractions. When bioaccessibility was measured, 6.8% of the samples in the <2 mm and <250 µm fractions, 2.3% in the <100 µm fraction, and 4.5% in the <63 µm fraction for adults showed an unacceptable level of risk. In children, the level of unacceptable risk was considerably higher: 90.9% in the <2 mm fraction, 93.2% in the <250 µm fraction, 84.1% in the <100 µm fraction, and 100% in the <63 µm fraction.

The results indicate that the hazard index values for systemic or non-cancer risks in adults and children were less than unity in all cases when bioaccessibility was taken into account, making the risk acceptable for the entire study area. However, when calculating the non-carcinogenic risks in the four granulometric fractions, 100% and 97% of the studied samples presented an unacceptable risk level for children and adults, respectively, in the absence of bioaccessibility.

In scenario E2, which pertains to the use of the area for tourism, Table 6 and Table 7 demonstrate that, when calculating the CDI and carcinogenic risk in the four granulometric fractions without bioaccessibility, 100% of the studied samples presented an unacceptable level of risk for both children and adults. However, when the bioaccessibility of these samples was considered, it was determined that there was no unacceptable risk for tourists, whether children or adults.

The systemic and non-carcinogenic risks for both age groups were evaluated. When bioaccessibility was included in the calculations, the hazard index was found to be below one in all cases, indicating that the risk was acceptable across the entire study area. However, when non-carcinogenic risks were calculated for the four particle size fractions without taking bioaccessibility into account, 97% of the studied samples showed an unacceptable level of risk for children.

In adults, the proportion of samples exhibiting an unacceptable risk was considerably lower. It was null in the <2 mm fraction, 2.3% in the <250 µm and 250–100 µm fractions, and 4.5% in the <63 µm fraction.

In Scenario E3 (agricultural use), for adults (see Table 6), when calculating the CDI and the carcinogenic risk in the four granulometric fractions without bioaccessibility, all of the studied samples presented an unacceptable level of risk. When bioaccessibility was considered, 6.8% of the samples in the <2 mm and <250 µm fractions and 4.5% in the <63 µm fraction exhibited an unacceptable level of risk.

The hazard index values for systemic or non-carcinogenic risks for both adults and children were below unity in all cases when bioaccessibility was taken into account, rendering the risk acceptable for the entire study area. When calculating the non-carcinogenic risk for the four granulometric fractions without bioaccessibility, it could be observed that 97% of the samples studied indicated an unacceptable risk level for adults.

### 3.5. Relationship Between the Variables Determined

#### 3.5.1. Relationship of the As Content with the Mineralogical Composition of the Soil

Following the verification of the data’s normality [Normality Test (Shapiro–Wilk) *p*-value > 0.05], a statistical analysis was conducted. This encompassed the assessment of correlations and the calculation of the Principal Component Analysis (PCA) for the total As, bioaccessibility, and mineralogy. A Pearson correlation matrix was made with the following levels of significance: [(*) *p* < 0.05 and (**) *p* < 0.001] for the studied samples. The total As content in the different fractions analysed exhibited a positive correlation with the phyllosilicates greenalite and clinochlore and a negative correlation with illite, aragonite, and calcite. Bioaccessible As demonstrated a positive correlation with the carbonates (aragonite and calcite), illite, and greenalite and a negative correlation with natrojarosite. High correlation coefficients are obtained between different minerals, which confirms the classification of these minerals into distinct groups. In particular, illite, aragonite, and calcite demonstrate positive correlations with each other whilst concurrently exhibiting negative correlations with jarosite and akaganeite. Table 8 shows the values of the correlation coefficients obtained for the most significant minerals.

A Principal Component Analysis (PCA) was conducted on the same variables, mineralogical composition, and total and bioaccessible As, selecting three factors that accounted for 73% of the variance. Factor 1 grouped variables common to the soils of zone 1: the wadi delta (feldspar, aragonite, calcite, and illite) and minerals of mining origin with a negative sign (natrojarosite, akaganeite, and clinochlore). Factor 2 was defined by minerals associated with the red soils that cross the wadi in the middle part, zone 4: grouping clinochlore, amphibole, and gypsum. Arsenic bioaccessibility was associated with goethite, greenalite, and the total content of the element in the positive part and jarosite in the negative part. Quartz behaved as a neutral variable, appearing with average scores in the three selected factors.

**Figure 8:**
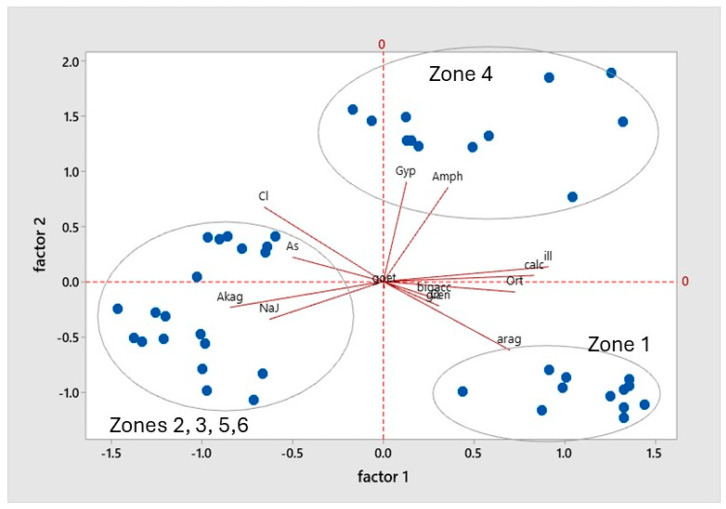
Circle of correlations between the mineralogical composition of the soil and the bioaccessible arsenic in the stomach (S.P.) and intestinal (I.P.) phases of digestion.

Figure 8 illustrates the distribution of the samples according to the factor scores obtained, thereby corroborating the aforementioned observations. The samples were grouped into three sets, which corresponded to samples from zone 4 (first quadrant) with amphibole and gypsum and samples from zone 1 (fourth quadrant). These samples had a medium total As content and a high content of bioaccessible As in the stomachal phase. They also contained aragonite in their mineralogical composition. The mineralogy of the samples was characterised by a high percentage of illite, greenalite, and quartz and a low content of akaganeite, calcite, and gypsum. The remainder of the samples were considered to be mining-influenced and contain natrojarosite.

**Figure 9:**
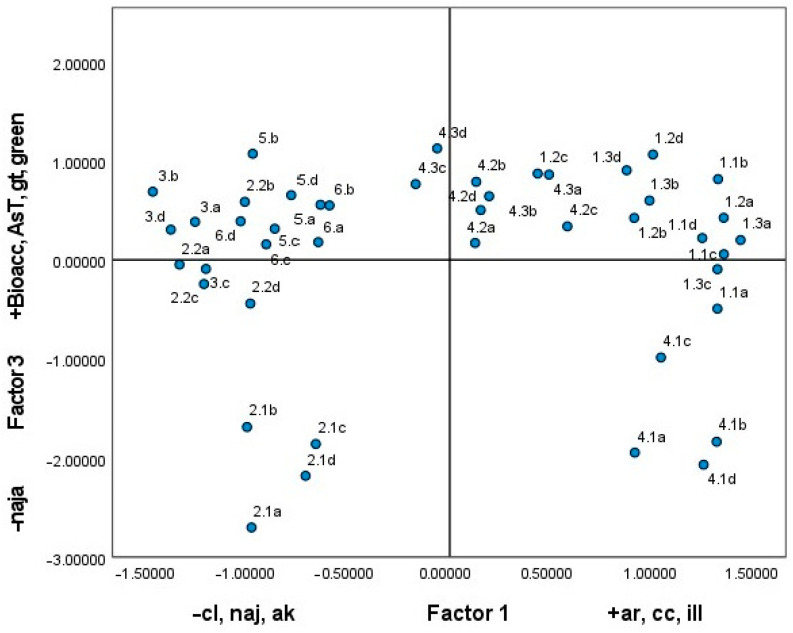
Principal Component Analysis (mineralogical composition and As content).

The graphical representation of the distribution of the samples against factors 1 and 3 (Figure 9) demonstrates the relationship between bioaccessibility and mineralogical composition.

The samples exhibiting higher bioaccessibility values, in relation to the carbonate and greenalite content, were situated in the upper part of the graph, within quadrants one and two. Conversely, the samples exhibiting lower bioaccessible As values, with a low total As content (sample 4.1) and high natrojarosite content (samples for zone 2), were situated in the lower part of the graph.

**Figure 10:**
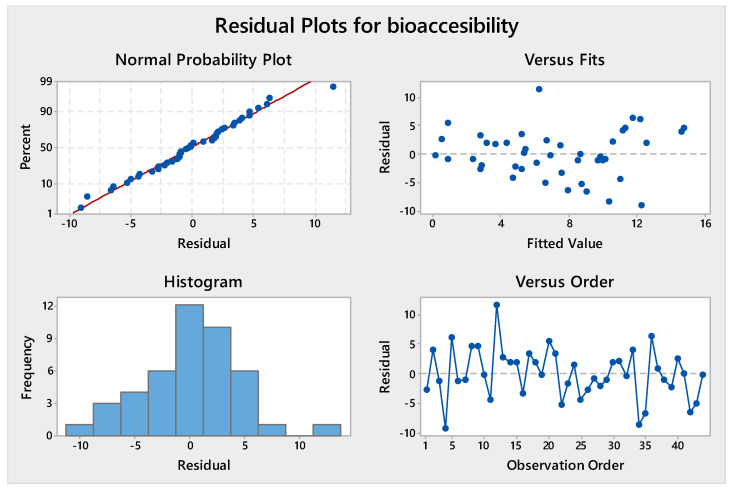
Graphically summarises the residual plots for bioaccesibility.

To identify a possible model that could simplify the relationship between bioaccessible arsenic (As) and the mineralogical composition of the samples in their different fractions, a linear regression was obtained. The linearity of the dependent and independent variables, and the homoscedasticity, independence, and normality of the residuals, were previously checked. Figure 10 graphically summarises the behaviour of the residuals obtained from the selected regression and shows that the results are acceptable.

This used bioaccessible As as the dependent variable, and the predictor variables were those with high coefficients and a low level of significance. Table 9 shows the values obtained for the linear regression, with a high correlation coefficient (0.719), the ANOVA analysis, and the coefficients obtained. The variable bioaccessible As was found to be positively related to the greenalite, illite, and As total content and negatively related to goethite. This regression does not include minerals that directly affect bioaccessibility, such as carbonates and jarosite, possibly because they are not normally distributed in the analysed samples.

The negative sign of goethite confirms its role in trapping bioaccessible As, which is retained in the crystal lattice. This preliminary model can be used to estimate the real risk associated with soil intake in different scenarios, provided the mineralogy is known and a total As determination is performed. Finally, the total As content is a positive component of the model, but its influence is reduced by the low value of its coefficient.

#### 3.5.2. Relationship Between the As Content of the Soil and the Risk Analysis Determined

The study identified a positive correlation between the total concentration of arsenic and the lifetime cancer risk and hazard in both children and adults across all land uses evaluated (residential, tourist, and agricultural). This implies that higher levels of As in soil pose a greater risk to human health, regardless of the fraction of soil studied. The risk analysis, which was calculated using the bioaccessible As concentration, indicates a positive but very low correlation in general. This correlation is practically insignificant in the finest soil fractions of <100 µm (R^2^ = 0.043; *p* < 0.05) and <63 µm (R^2^ = 0.049; *p* < 0.05). It is, however, important to note the positive and high correlation of R^2^ = 0.807; *p* < 0.05 between bioaccessible arsenic and the <63 µm fraction in the carcinogenic risk analysis in children for residential soil use.

For the coarser granulometric fractions, there is a positive correlation between bioaccessible As and the respective fractions. The correlation coefficients are 0.335 and 0.253, respectively, with *p*-values less than 0.05.

## 4. Conclusions

This study demonstrates the importance of the Beal wadi in understanding the processes related to geogenic arsenic contamination, enabling the prioritisation of risk zones. It also identifies the primary materials in the area’s soils and their mineralogical characteristics. The wadi represents an excellent scenario to understand the processes linked to the transport and fate of As, contemplating all forms: soluble As, particulate As, and mineralogical species, as well as its bioaccessibility to humans.

This case study provides insight into the wider impact on other CAMAS affected by temporary watercourses and semi-arid climatic conditions. It also enables us to expand our understanding of the actual issues surrounding As transfer. Consideration of Los Nietos Beach, a neighbouring site to the study area, reveals that the mineralogy and As content analysed are not equivalent to those in sampling zones 1 and 2. This is consistent with the conditions of the coastal dynamics [68] and indicates that the influence of geogenic As is greatly diluted by contributions from neighbouring agricultural soils. Nevertheless, bioaccessibility studies are necessary for correctly interpreting the risk posed by these bordering beach sands.

The mean total arsenic concentration was highest in the smallest soil particle size fraction (<63 µm). All four particle size fractions contained the same minerals, albeit in different proportions. Further research is required to determine the significance of mineralogical composition in relation to the contribution of minerals to bioaccessible arsenic content. Since arsenic is most soluble in acidic pH, specifically during the stomach phase of digestion, the <250 µm fraction is the most appropriate for health risk analysis studies. The study concludes that the bioaccessible As content in the <63 µm fraction is a crucial indicator for risk analysis in children when dealing with residential land use in areas affected by past mining activities. Consequently, the residential use of these soils in the study area is discouraged for children.

In studies analysing human health risks, it is crucial to determine the bioaccessible arsenic content. The total concentration in soil is not a reliable indicator of the bioaccessible fraction, which can lead to serious errors and unnecessary social alarm. Without considering the bioaccessible As fraction, assessing the potential risk can result in an overestimation of both systemic and carcinogenic risks.

The presence of organic matter and the reductive conditions that are often associated with it represents a significant risk to both human health and the integrity of ecosystems. This is due to the fact that these conditions favour the presence of reduced forms of arsenic that are highly toxic.

The marine sediments of Los Nietos Beach did not show a mining influence due to the transport of materials from the Beal wadi.

## Figures and Tables

**Table 1:** Parameters used to calculate the Chemical Daily Intake in present day scenarios.

		Adults (Use)			Children (Use)	
Variable		E1 Residential	E2 Tourism	E3 Agriculture	E1 Residential	E2 Tourism
**Cs**: arsenic concentration soil. (mg/kg). Total/bioaccessible		-	-	-	-	-
**IR**: Soil ingestion rate (mg/day)		100	100	200	200	200
**EF**: Exposure frequency (days)		350	60	150	350	60
**ED**: exposure duration (years)		30	30	30	6	6
**CF**: Conversion Factor (dimensionless)		1.10^−6^	1.10^−6^	1.10^−6^	1.10^−6^	1.10^−6^
**FI**: ingestion factor (dimensionless)		1	1	1	1	1
**BW**: body weight (Kg)		70	70	70	15	15
AT: averaging time (days)	Carcinogenic	25550	25550	25550	25550	25550
	Hazard index	2190.ED	10950.ED	10950.ED	2190.ED	2190.ED

**Table 2:** Summary of the toxicological information of arsenic.

**Arsenic**	**Non-Carcinogen Toxicity**		**Carcinogen Toxicity**	
	**RfD Oral** **(mg/kg·day)**		**SF Oral** **(mg/kg·day)**	
	0.0003	EPA	1.5	EPA

The non-carcinogenic constants, in the absence of official data, are established by route–route extrapolation according to the EPA procedure. Contaminated Soil Guide 2017 Junta de Andalucía.

**Table 3:** General characteristics of the soils.

Samples	pH	E.C. (mS/cm)	O.M. (%)	Texture (100%)		
				Clay	Silt	Sand
**1.1**	7.6	10.0	2.5	1.8	45.3	52.9
**1.2**	7.3	25.8	2.9	2.5	41.1	56.4
**1.3**	7.7	10.0	1.6	4.8	28.2	67
**2.1**	2.8	16.6	N.D.	22.3	57.5	20.2
**2.2**	3.3	21.8	N.D.	20.9	52.6	26.5
**3**	4.4	2.2	N.D.	5.5	18.1	76.4
**4.1**	5.4	3.1	N.D.	6.2	29.7	64.1
**4.2**	6.1	2.2	N.D.	4.9	25.2	69.9
**4.3**	5.2	2.5	N.D.	65.2	28.6	6.2
**5**	5.4	1.9	N.D.	3.1	8.4	88.5
**6**	4.6	2.14	N.D.	1.5	12.8	85.7
**Mean ± SD**	5.4 ± 1.6	8.9 ± 8.8	-	12.4 ± 18.9	26.9 ± 15.4	6.06 ± 28.8

E.C.: electrical conductivity; O.M.: organic matter; SD: standard deviation; N.D.: not detected.

**Table 4:** Arsenic content.

[As] mg/kg												
Samples	Total < 2 mm	Stomach Phase < 2 mm	Intestinal Phase < 2 mm	Total < 250 µm	Stomach Phase < 250 µm	Intestinal Phase < 250 µm	Total < 100 µm	Stomach Phase < 100 µm	Intestinal Phase < 100 µm	Total < 63 µm	Stomach Phase < 63 µm	Intestinal Phase < 63 µm
**1.1**	489	2.5	1.5	580	3.2	1.9	495	8.6	<ld	644	18.6	3.5
**1.2**	539	18.3	<ld	809	19.5	<ld	845	7.5	<ld	728	8.8	0.7
**1.3**	528	16.1	<ld	611	17.8	<ld	690	6.7	<ld	712	8.6	<ld
**2.1**	283	3.2	<ld	1269	4.3	<ld	339	5.1	<ld	395	6.3	2.4
**2.2**	1184	6.2	<ld	1070	6.3	<ld	880	<ld	<ld	1199	5.5	1.2
**3**	959	8.7	<ld	853	9.1	<ld	1055	4.5	1.64	1040	3.5	1.9
**4.1**	75	0.5	<ld	95	0.9	<ld	90	<ld	<ld	85	<ld	<ld
**4.2**	1072	9.2	<ld	699	9.4	<ld	1135	12.8	3.60	1337	14.6	3.5
**4.3**	1230	15.3	<ld	1314	18.1	<ld	1364	1.66	2.49	740	1.9	<ld
**5**	945	6.4	<ld	1089	9.2	<ld	1204	2.65	<ld	1214	0.9	1.4
**6**	845	5.5	<ld	1199	6.7	<ld	1230	1.62	<ld	1380	0.5	1.6
**Range**	75–1184	0.5–18.3	<0.5–1.5	95–1314	0.9–19.5	<0.5–1.9	90–1364	<0.5–12.8	<0.5–3.6	85–1380	0.5–18.6	0.7–3.5
**Media ± SD**	740.8 ± 380.5	8.3 ± 6.1	-	871.6 ± 365.7	9.5 ± 6.3	-	847.9 ± 408.1	4.7 ± 3.9	2.6 ± 0.9	861.3 ± 408.8	6.3 ± 6.0	1.6 ± 1.2

SD: Standard deviation; ld = 0.5.

**Table 5:** Runoff water characteristics.

Sample	pH	Eh	E.C.	[As] μg L^−1^		
		mV	(mS/cm)	[As] Total	%[As] III	%[As] V
**w-1**	7.03 ± 0.56	410 ± 9	14.35 ± 0.09	15 ± 6	2	98
**w-2**	4.39 ± 1.51	310 ± 6	12.56 ± 0.18	950 ± 320	4	96
**w-3**	8.98 ± 0.75	430 ± 8	3.54 ± 0.17	5 ± 2	1	99

**Table 6:** Risk characterisation for As for children.

Risk Characterisation for As for Children																
Scenario E1: Residential Use																
	<2 mm				<250 µm				<100 µm				<63 µm			
**SAMPLES**	Without bioaccessibility		With bioaccessibility		Without bioaccessibility		With bioaccessibility		Without bioaccessibility		With bioaccessibility		Without bioaccessibility		With bioaccessibility	
	Carcinogenic risk	Hazard index	Carcinogenic Risk	Hazard index	Carcinogenic risk	Hazard index	Carcinogenic risk	Hazard index	Carcinogenic risk	Hazard index	Carcinogenic risk	Hazard index	Carcinogenic risk	Hazard index	Carcinogenic risk	Hazard index
**1.1**	**8.0 × 10^−4^**	**20.84**	4.1 × 10^−6^	0.11	**9.5 × 10^−4^**	**24.72**	5.3 × 10^−6^	0.14	**8.1 × 10^−4^**	**21.10**	**1.4 × 10^−5^**	0.37	**1.1 × 10^−3^**	**27.45**	**3.1 × 10^−5^**	0.79
**1.2**	**8.9 × 10^−4^**	**22.97**	**3.0 × 10^−5^**	0.78	**1.3 × 10^−3^**	**34.48**	**3.2 × 10^−5^**	0.83	**1.4 × 10^−3^**	**36.01**	**1.2 × 10^−5^**	0.32	**1.2 × 10^−3^**	**31.03**	**1.4 × 10^−5^**	0.37
**1.3**	**8.7 × 10^−4^**	**22.50**	**2.6 × 10^−5^**	0.69	**1.0 × 10^−3^**	**26.04**	**2.9 × 10^−5^**	0.76	**1.1 × 10^−3^**	**29.41**	**1.1 × 10^−5^**	0.29	**1.2 × 10^−3^**	**30.34**	**1.4 × 10^−5^**	0.37
**2.1**	**4.7 × 10^−4^**	**12.06**	5.3 × 10^−6^	0.14	**2.1 × 10^−3^**	**54.08**	7.1 × 10^−6^	0.18	**5.6 × 10^−4^**	**14.45**	8.4 × 10^−6^	0.22	**6.5 × 10^−4^**	**16.83**	**1.0 × 10^−5^**	0.27
**2.2**	**1.9 × 10^−3^**	**50.46**	**1.0 × 10^−5^**	0.26	**1.8 × 10^−3^**	**45.60**	**1.0 × 10^−5^**	0.27	**1.4 × 10^−3^**	**37.50**	6.6 × 10^−7^	0.02	**2.0 × 10^−3^**	**51.10**	9.1 × 10^−6^	0.23
**3**	**1.6 × 10^−3^**	**40.87**	**1.4 × 10^−5^**	0.37	**1.4 × 10^−3^**	**36.35**	**1.5 × 10^−5^**	0.39	**1.7 × 10^−3^**	**44.96**	7.4 × 10^−6^	0.19	**1.7 × 10^−3^**	**44.32**	5.8 × 10^−6^	0.15
**4.1**	**1.2 × 10^−4^**	**3.20**	8.2 × 10^−7^	0.02	**1.6 × 10^−4^**	**4.05**	1.5 × 10^−6^	0.04	**1.5 × 10^−4^**	**3.84**	6.6 × 10^−8^	0.00	**1.4 × 10^−4^**	**3.62**	3.3 × 10^−8^	0.00
**4.2**	**1.8 × 10^−3^**	**45.69**	**1.5 × 10^−5^**	0.39	**1.1 × 10^−3^**	**29.79**	**1.5 × 10^−5^**	0.40	**1.9 × 10^−3^**	**48.37**	**2.1 × 10^−5^**	0.55	**2.2 × 10^−3^**	**56.98**	**2.4 × 10^−5^**	0.62
**4.3**	**2.0 × 10^−3^**	**52.42**	**2.5 × 10^−5^**	0.65	**2.2 × 10^−3^**	**56.00**	**3.0 × 10^−5^**	0.77	**2.3 × 10^−3^**	**58.81**	4.1 × 10^−6^	0.11	**1.2 × 10^−3^**	**31.54**	3.1 × 10^−6^	0.08
**5**	**1.6 × 10^−3^**	**40.27**	**1.1 × 10^−5^**	0.27	**1.8 × 10^−3^**	**46.41**	**1.5 × 10^−5^**	0.39	**2.0 × 10^−3^**	**51.31**	4.4 × 10^−6^	0.11	**2.0 × 10^−3^**	**51.74**	2.3 × 10^−6^	0.06
**6**	**1.4 × 10^−3^**	**36.01**	9.0 × 10^−6^	0.23	**2.0 × 10^−3^**	**51.10**	**1.1 × 10^−5^**	0.29	**2.0 × 10^−3^**	**52.42**	2.7 × 10^−6^	0.07	**2.2 × 10^−3^**	**58.13**	2.7 × 10^−6^	0.07
**Scenario E2: Tourism Use**																
**1.1**	**1.4 × 10^−4^**	**3.57**	7.0 × 10^−7^	0.02	**1.6 × 10^−4^**	**4.24**	9.0 × 10^−7^	0.02	**1.4 × 10^−4^**	**3.62**	2.4 × 10^−6^	0.06	**1.8 × 10^−4^**	**4.71**	5.2 × 10^−6^	0.14
**1.2**	**1.5 × 10^−4^**	**3.94**	5.2 × 10^−6^	0.13	**2.3 × 10^−4^**	**5.91**	5.5 × 10^−6^	0.14	**2.4 × 10^−4^**	**6.17**	2.1 × 10^−6^	0.05	**2.1 × 10^−4^**	**5.32**	2.5 × 10^−6^	0.06
**1.3**	**1.5 × 10^−4^**	**3.86**	4.5 × 10^−6^	0.12	**1.7 × 10^−4^**	**4.46**	5.0 × 10^−6^	0.13	**1.9 × 10^−4^**	**5.04**	1.9 × 10^−6^	0.05	**2.0 × 10^−4^**	**5.20**	2.4 × 10^−6^	0.06
**2.1**	**8.0 × 10^−5^**	**2.07**	9.0 × 10^−7^	0.02	**3.6 × 10^−4^**	**9.27**	1.2 × 10^−6^	0.03	**9.6 × 10^−5^**	**2.48**	1.4 × 10^−6^	0.04	**1.1 × 10^−4^**	**2.89**	1.8 × 10^−6^	0.05
**2.2**	**3.3 × 10^−4^**	**8.65**	1.7 × 10^−6^	0.05	**3.0 × 10^−4^**	**7.82**	1.8 × 10^−6^	0.05	**2.5 × 10^−4^**	**6.43**	1.1 × 10^−7^	0.00	**3.4 × 10^−4^**	**8.76**	1.6 × 10^−6^	0.04
**3**	**2.7 × 10^−4^**	**7.01**	2.5 × 10^−6^	0.06	**2.4 × 10^−4^**	**6.23**	2.6 × 10^−6^	0.07	**3.0 × 10^−4^**	**7.71**	1.3 × 10^−6^	0.03	**2.9 × 10^−4^**	**7.60**	9.9 × 10^−7^	0.03
**4.1**	**2.1 × 10^−5^**	**0.55**	1.4 × 10^−7^	0.00	**2.7 × 10^−5^**	0.69	2.5 × 10^−7^	0.01	**2.5 × 10^−5^**	0.66	1.1 × 10^−8^	0.00	**2.4 × 10^−5^**	0.62	5.6 × 10^−9^	0.00
**4.2**	**3.0 × 10^−4^**	**7.83**	2.6 × 10^−6^	0.07	**2.0 × 10^−4^**	**5.11**	2.6 × 10^−6^	0.07	**3.2 × 10^−4^**	**8.29**	3.6 × 10^−6^	0.09	**3.8 × 10^−4^**	**9.77**	4.1 × 10^−6^	0.11
**4.3**	**3.5 × 10^−4^**	**8.99**	4.3 × 10^−6^	0.11	**3.7 × 10^−4^**	**9.60**	5.1 × 10^−6^	0.13	**3.9 × 10^−4^**	**10.08**	7.0 × 10^−7^	0.02	**2.1 × 10^−4^**	**5.41**	5.3 × 10^−7^	0.01
**5**	**2.7 × 10^−4^**	**6.90**	1.8 × 10^−6^	0.05	**3.1 × 10^−4^**	**7.96**	2.6 × 10^−6^	0.07	**3.4 × 10^−4^**	**8.80**	7.5 × 10^−7^	0.02	**3.4 × 10^−4^**	**8.87**	4.0 × 10^−7^	0.01
**6**	**2.4 × 10^−4^**	**6.17**	1.5 × 10^−6^	0.04	**3.4 × 10^−4^**	**8.76**	1.9 × 10^−6^	0.05	**3.5 × 10^−4^**	**8.99**	4.6 × 10^−7^	0.01	**3.8 × 10^−4^**	**9.97**	4.6 × 10^−7^	0.01

**Risk > 10^−5^** unacceptable health risk. **HI > 1** unacceptable health risk.

**Table 7:** Risk characterisation for As for adults.

Risk Characterisation for As for Adults																
Scenario E1: Residential Use																
	<2 mm				<250 µm				<100 µm				<63 µm			
**SAMPLES**	Without bioaccessibility		With bioaccessibility		Without bioaccessibility		With bioaccessibility		Without bioaccessibility		With bioaccessibility		Without bioaccessibility		With bioaccessibility	
	Carcinogenic risk	Hazard index	Carcinogenic Risk	Hazard index	Carcinogenic risk	Hazard Index	Carcinogenic risk	Hazard index	Carcinogenic risk	Hazard index	Carcinogenic risk	Hazard index	Carcinogenic risk	Hazard index	Carcinogenic risk	Hazard index
**1.1**	**4.3 × 10^−4^**	**2.23**	2.2 × 10^−6^	0.01	**5.1 × 10^−4^**	**2.65**	2.8 × 10^−6^	0.01	**4.4 × 10^−4^**	**2.26**	7.6 × 10^−6^	0.04	**5.7 × 10^−4^**	**2.94**	**1.6 × 10^−5^**	0.08
**1.2**	**4.7 × 10^−4^**	**2.46**	**1.6 × 10^−5^**	0.08	**7.1 × 10^−4^**	**3.69**	**1.7 × 10^−5^**	0.09	**7.4 × 10^−4^**	**3.86**	6.6 × 10^−6^	0.03	**6.4 × 10^−4^**	**3.32**	7.7 × 10^−6^	0.04
**1.3**	**4.6 × 10^−4^**	**2.41**	**1.4 × 10^−5^**	0.07	**5.4 × 10^−4^**	**2.79**	**1.6 × 10^−5^**	0.08	**6.1 × 10^−4^**	**3.15**	5.9 × 10^−6^	0.03	**6.3 × 10^−4^**	**3.25**	7.6 × 10^−6^	0.04
**2.1**	**2.5 × 10^−4^**	**1.29**	2.8 × 10^−6^	0.01	**1.1 × 10^−3^**	**5.79**	3.8 × 10^−6^	0.02	**3.0 × 10^−4^**	**1.55**	4.5 × 10^−6^	0.02	**3.5 × 10^−4^**	**1.80**	5.6 × 10^−6^	0.03
**2.2**	**1.0 × 10^−3^**	**5.41**	5.5 × 10^−6^	0.03	**9.4 × 10^−4^**	**4.89**	5.5 × 10^−6^	0.03	**7.7 × 10^−4^**	**4.02**	3.5 × 10^−7^	0.00	**1.1 × 10^−3^**	**5.47**	4.9 × 10^−6^	0.03
**3**	**8.4 × 10^−4^**	**4.38**	7.7 × 10^−6^	0.04	**7.5 × 10^−4^**	**3.89**	8.0 × 10^−6^	0.04	**9.3 × 10^−4^**	**4.82**	4.0 × 10^−6^	0.02	**9.2 × 10^−4^**	**4.75**	3.1 × 10^−6^	0.02
**4.1**	**6.6 × 10^−5^**	0.34	4.4 × 10^−7^	0.00	**8.4 × 10^−5^**	0.43	7.9 × 10^−7^	0.00	**7.9 × 10^−5^**	0.41	3.5 × 10^−8^	0.00	**7.5 × 10^−5^**	0.39	1.8 × 10^−8^	0.00
**4.2**	**9.4 × 10^−4^**	**4.89**	8.1 × 10^−6^	0.04	**6.2 × 10^−4^**	**3.19**	8.3 × 10^−6^	0.05	**1.0 × 10^−3^**	**5.18**	**1.1 × 10^−5^**	0.06	**1.2 × 10^−3^**	**6.11**	**1.3 × 10^−5^**	0.07
**4.3**	**1.1 × 10^−3^**	**5.62**	**1.3 × 10^−5^**	0.07	**1.2 × 10^−3^**	**6.00**	**1.6 × 10^−5^**	0.08	**1.2 × 10^−3^**	**6.30**	2.2 × 10^−6^	0.01	**6.5 × 10^−4^**	**3.38**	1.7 × 10^−6^	0.01
**5**	**8.3 × 10^−4^**	**4.32**	5.6 × 10^−6^	0.03	**9.6 × 10^−4^**	**4.97**	8.1 × 10^−6^	0.04	**1.1 × 10^−3^**	**5.50**	2.3 × 10^−6^	0.01	**1.1 × 10^−3^**	**5.54**	1.2 × 10^−6^	0.01
**6**	**7.4 × 10^−4^**	**3.86**	4.8 × 10^−6^	0.03	**1.1 × 10^−3^**	**5.47**	5.9 × 10^−6^	0.03	**1.1 × 10^−3^**	**5.62**	1.4 × 10^−6^	0.01	**1.2 × 10^−3^**	**6.23**	1.4 × 10^−6^	0.01
**Scenario E2: tourism use**																
**1.1**	**7.4 × 10^−5^**	0.38	3.8 × 10^−7^	0.00	**8.8 × 10^−5^**	0.45	4.8 × 10^−7^	0.00	**7.5 × 10^−5^**	0.39	1.3 × 10^−6^	0.01	**9.7 × 10^−5^**	0.50	2.8 × 10^−6^	0.01
**1.2**	**8.1 × 10^−5^**	0.42	2.8 × 10^−6^	0.01	**1.2 × 10^−4^**	0.63	2.9 × 10^−6^	0.02	**1.3 × 10^−4^**	0.66	1.1 × 10^−6^	0.01	**1.1 × 10^−4^**	0.57	1.3 × 10^−6^	0.01
**1.3**	**8.0 × 10^−5^**	0.41	2.4 × 10^−6^	0.01	**9.2 × 10^−5^**	0.48	2.7 × 10^−6^	0.01	**1.0 × 10^−4^**	0.54	1.0 × 10^−6^	0.01	**1.1 × 10^−4^**	0.56	1.3 × 10^−6^	0.01
**2.1**	**4.3 × 10^−5^**	0.22	4.8 × 10^−7^	0.00	**1.9 × 10^−4^**	0.99	6.5 × 10^−7^	0.00	**5.1 × 10^−5^**	0.27	7.7 × 10^−7^	0.00	**6.0 × 10^−5^**	0.31	9.5 × 10^−7^	0.00
**2.2**	**1.8 × 10^−4^**	0.93	9.4 × 10^−7^	0.00	**1.6 × 10^−4^**	0.84	9.5 × 10^−7^	0.00	**1.3 × 10^−4^**	0.69	6.0 × 10^−8^	0.00	**1.8 × 10^−4^**	0.94	8.3 × 10^−7^	0.00
**3**	**1.4 × 10^−4^**	0.75	1.3 × 10^−6^	0.01	**1.3 × 10^−4^**	0.67	1.4 × 10^−6^	0.01	**1.6 × 10^−4^**	0.83	6.8 × 10^−7^	0.00	**1.6 × 10^−4^**	0.81	5.3 × 10^−7^	0.00
**4.1**	**1.1 × 10^−5^**	0.06	7.5 × 10^−8^	0.00	**1.4 × 10^−4^**	0.07	1.4 × 10^−7^	0.00	**1.4 × 10^−5^**	0.07	6.0 × 10^−9^	0.00	**1.3 × 10^−5^**	0.07	3.0 × 10^−9^	0.00
**4.2**	**1.6 × 10^−4^**	0.84	1.4 × 10^−6^	0.01	**1.1 × 10^−4^**	0.55	1.4 × 10^−6^	0.01	**1.7 × 10^−4^**	0.89	1.9 × 10^−6^	0.01	**2.0 × 10^−4^**	**1.05**	2.2 × 10^−6^	0.01
**4.3**	**1.9 × 10^−4^**	0.96	2.3 × 10^−6^	0.01	**2.0 × 10^−4^**	**1.03**	2.7 × 10^−6^	0.01	**2.1 × 10^−4^**	**1.08**	3.8 × 10^−7^	0.00	**1.1 × 10^−4^**	0.58	2.8 × 10^−7^	0.00
**5**	**1.4 × 10^−4^**	0.74	9.7 × 10^−7^	0.01	**1.6 × 10^−4^**	0.85	1.4 × 10^−6^	0.01	**1.8 × 10^−4^**	0.94	4.0 × 10^−7^	0.00	**1.8 × 10^−4^**	0.95	2.1 × 10^−7^	0.00
**6**	**1.3 × 10^−4^**	0.66	8.3 × 10^−7^	0.00	**1.8 × 10^−4^**	0.94	1.0 × 10^−6^	0.01	**1.9 × 10^−4^**	0.96	2.4 × 10^−7^	0.00	**2.1 × 10^−4^**	**1.07**	2.5 × 10^−7^	0.00
**Scenario E3: agriculture use**																
**1.1**	**3.7 × 10^−4^**	**1.91**	1.9 × 10^−6^	0.01	**4.4 × 10^−4^**	**2.27**	2.4 × 10^−6^	0.01	**3.7 × 10^−4^**	**1.94**	6.5 × 10^−6^	0.03	**4.9 × 10^−4^**	**2.52**	**1.4 × 10^−5^**	0.07
**1.2**	**4.1 × 10^−4^**	**2.11**	**1.4 × 10^−5^**	0.07	**6.1 × 10^−4^**	**3.17**	**1.5 × 10^−5^**	0.07	**6.4 × 10^−4^**	**3.31**	5.7 × 10^−6^	0.03	**5.5 × 10^−4^**	**2.85**	6.6 × 10^−6^	0.03
**1.3**	**4.0 × 10^−4^**	**2.07**	**1.2 × 10^−5^**	0.06	**4.6 × 10^−4^**	**2.39**	**1.3 × 10^−5^**	0.07	**5.2 × 10^−4^**	**2.70**	5.1 × 10^−6^	0.03	**5.4 × 10^−4^**	**2.79**	6.5 × 10^−6^	0.03
**2.1**	**2.1 × 10^−4^**	**1.11**	2.4 × 10^−6^	0.01	**9.6 × 10^−4^**	**4.97**	3.2 × 10^−6^	0.02	**2.6 × 10^−4^**	**1.33**	3.8 × 10^−6^	0.02	**3.0 × 10^−4^**	**1.55**	4.8 × 10^−6^	0.02
**2.2**	**8.9 × 10^−4^**	**4.63**	4.7 × 10^−6^	0.02	**8.1 × 10^−4^**	**4.19**	4.8 × 10^−6^	0.02	**6.6 × 10^−4^**	**3.44**	3.0 × 10^−7^	0.00	**9.1 × 10^−4^**	**4.69**	4.2 × 10^−6^	0.02
**3**	**7.2 × 10^−4^**	**3.75**	6.6 × 10^−6^	0.03	**6.4 × 10^−4^**	**3.34**	6.9 × 10^−6^	0.03	**8.0 × 10^−4^**	**4.13**	3.4 × 10^−6^	0.02	**7.9 × 10^−4^**	**4.07**	2.6 × 10^−6^	0.01
**4.1**	**5.7 × 10^−5^**	0.29	3.8 × 10^−7^	0.00	**7.2 × 10^−5^**	0.37	6.8 × 10^−7^	0.00	**6.8 × 10^−5^**	0.35	3.0 × 10^−8^	0.00	**6.4 × 10^−5^**	0.33	1.5 × 10^−8^	0.00
**4.2**	**8.1 × 10^−4^**	**4.20**	6.9 × 10^−6^	0.04	**5.3 × 10^−4^**	**2.74**	7.1 × 10^−6^	0.04	**8.6 × 10^−4^**	**4.44**	9.7 × 10^−6^	0.05	**1.0 × 10^−3^**	**5.23**	**1.1 × 10^−5^**	0.06
**4.3**	**9.3 × 10^−4^**	**4.81**	**1.2 × 10^−5^**	0.06	**9.9 × 10^−4^**	**5.14**	**1.4 × 10^−5^**	0.07	**1.0 × 10^−3^**	**5.40**	1.9 × 10^−6^	0.01	**5.6 × 10^−4^**	**2.90**	1.4 × 10^−6^	0.01
**5**	**7.1 × 10^−4^**	**3.70**	4.8 × 10^−6^	0.03	**8.2 × 10^−4^**	**4.26**	6.9 × 10^−6^	0.04	**9.1 × 10^−4^**	**4.71**	2.0 × 10^−6^	0.01	**9.2 × 10^−4^**	**4.75**	1.1 × 10^−6^	0.01
**6**	**6.4 × 10^−4^**	**3.31**	4.2 × 10^−6^	0.02	**9.1 × 10^−4^**	**4.69**	5.1 × 10^−6^	0.03	**9.3 × 10^−4^**	**4.81**	1.2 × 10^−6^	0.01	**1.0 × 10^−3^**	**5.34**	1.2 × 10^−6^	0.01

**Risk > 10^−5^** unacceptable health risk. **HI > 1** unacceptable health risk.

**Table 8:** Values of the correlation coefficients for the minerals.

	arag	calc	ill	gren	Cl	NaJ	akag	As	bioac
Q	0.492 **	0.276	0.182	0.330 *	−0.336 *	−0.544 **	−0.183	0.095	0.276
ort	0.539 **	0.620 **	0.656 **	0.333 *	−0.479 **	−0.527 **	−0.476 **	0.204	0.290
arag		0.621 **	0.566 **	0.499 **	−0.835 **	−0.387 **	−0.455 **	0.300 *	0.402 **
calc			0.740 **	0.467 **	−0.421 **	−0.688 **	−0.766 **	0.155	0.302 *
ill				0.346 *	−0.492 **	−0.687 **	−0.781 **	0.305 *	0.392 **
gren					−0.188	−0.513 **	−0.298 *	0.321 *	0.563 **
Cl						0.115	0.373 *	0.583 **	−0.260
NaJ							0.500 **	0.160	−0.293

Q = quartz; ort = feldspar; arag = aragonite; calc = calcite; ill = illite; gren = greenalite; Cl = clinochlore; NaJ = natrojarosite; akag = akageneite; As = As total content; bioac = bioaccesibility.

**Table 9:** Model summary for As bioaccessibility.

**Model Summary**	**R**	**R Squared**	**Adjusted R Squared**	**Std. Error of the Estimate**	**Sig.**
	**0.713**	**0.509**	**0.458**	**3.89805**	
	Unstandardised Coefficients		Standardised Coefficients	t	
	B	Std. Error	Beta		
(Constant)	−2.812	2.624		−1.072	0.290
illite	0.184	0.079	0.314	2.315	0.026
greenalite	0.752	0.214	0.484	3.515	0.01
As total content	0.004	0.002	0.290	2.116	0.041
goethite	−0.350	0.168	−0.249	−2.088	0.43
ANOVA Model Summary Regression Residual Total	Sum of squares	gl	Mean quadratic	F	Sig.
	613.639	4	153.410	10.096	0.000
	592.596	39	15.195		
	1206.235	43			

## Data Availability

Data will be made available on request.
